# Field-based evaluation of trunk strength using a novel in-chair assessment system in elite wheelchair athletes

**DOI:** 10.1038/s41598-026-44257-2

**Published:** 2026-03-12

**Authors:** Kirill Schaaf, Maurice Nquiti, Paula Lassner, Käthe Bersiner, Sebastian Gehlert, Thomas Abel, Daniel Jacko

**Affiliations:** 1 Department of Molecular and Cellular Sports Medicine, Institute of Cardiovascular Research and Sports Medicine, German Sport University Cologne, Am Sportpark Müngersdorf 6, 50933 Cologne, Germany; 2 Olympic Base Center North Rhine-Westfalia/Rhineland, Cologne, Germany; 3 Department for the Biosciences of Sports, Institute of Sports Science, University of Hildesheim, Hildesheim, Germany; 4 Institute of Movement and Neuroscience, German Sport University Cologne, Cologne, Germany

**Keywords:** Trunk strength, Wheelchair basketball, Wheelchair sports, Isometric strength testing, Functional classification, Reliability and validity, Anatomy, Engineering, Health care, Medical research

## Abstract

Trunk strength is essential for performance in wheelchair-based sports, yet assessment under sport-specific conditions is challenging. The athlete and their sport wheelchair act as a unit, so meaningful assessment should occur in the wheelchair itself. We developed a mobile device that measures isometric trunk flexion, extension, and lateral flexion directly in the athlete’s own sport wheelchair. We first examined test-retest reliability in both able-bodied participants and elite wheelchair basketball (WB) players. Both cohorts showed high day-to-day consistency (intraclass correlation coefficient, 0.85–0.97; coefficient of variation, < 10%). We then demonstrated that trunk forces measured distinguish between established WB functional classes 1.0 (high impairment) – 4,5 (low impairment). Higher classified groups produced significantly greater forces, with a strong positive correlation between classification and force (*r* = 0.76–0.87). Next, classification-specific reference values were generated and compared with measures from able-bodied participants. Lower classified athletes (1.0-3.5) produced approximately 56% less trunk force than able-bodied participants, whereas players in the highest classification (4.0-4.5) demonstrated comparable or up to 31% higher force in trunk flexion. These findings support the device as a reliable, field-based method to quantify trunk strength in athletes’ own wheelchairs and show that it can differentiate functional classes, enabling its application in performance testing.

## Introduction

Trunk strength is considered an important factor of performance and impairment classification in wheelchair-based sports, particularly in wheelchair basketball (WB)^1–6^. However, sport-specific trunk strength diagnostics that are portable, chair-integrated, and capable of being applied across a range of impairments remain scarce^6–8^. Trunk strength and trunk impairment have been assessed using controlled, laboratory-based dynamometry^9^ or related objective measurement set-ups^10^, which are limited by their transportability and typically do not replicate the athlete-wheelchair unit that fundamentally shapes movement strategies in wheelchair sports^6,7^. This creates a methodological gap that could be addressed by a field-deployable device that quantifies trunk strength in the athlete’s own wheelchair. Such a device could improve ecological validity, support routine performance diagnostics, and provide objective information relevant to classification-related assessments^1,2,5,11^.

From a biomechanical perspective, trunk strength contributes to spinal stability^12^, enables efficient force transmission between the upper and the lower body^13^, and is linked to performance-relevant functions such as balance^14^ as well as injury prevention^15^. In able-bodied sports trunk-strength is widely considered relevant to athletic performance and is therefore commonly trained and assessed in performance settings^16–19^.

In wheelchair-based sports, trunk strength is of particular relevance. Here, the trunk plays a central role in the biomechanical link between force generation in the upper limbs and the sport chair. Studies using electromyography confirm the extensive involvement of the trunk muscles during chair propulsion^20^ and high-intensity tasks, such as sprinting or acceleration in racing settings^21^. Furthermore, trunk kinematics contribute to propulsion speed and performance beyond upper limb movement alone^21,22^. Importantly, trunk control, which is related to objective measures of trunk impairment^10^, is a central construct in evidence-based classification discussions^1,2^ and is associated with performance outcomes in wheelchair-based sports like basketball^5,6^ and rugby^4,6^. Particularly in wheelchair basketball, trunk strength significantly influences performance, as athletes with limited trunk control may be restricted in terms of acceleration-related tasks and stability demands during typical game situations (e.g., external perturbations/contact), consistent with evidence linking trunk impairment to wheelchair court-sport performance^3,5,6^ and propulsion/start performance^1,2^.

Despite its high relevance, reliable and sport-specific diagnostic methods for evaluation of trunk strength of wheelchair athletes remain challenging. While conventional, laboratory based diagnostic devices like the IsoMed or the Biodex dynamometer allow for reliable isometric and isokinetic measurements^9,23^, they also have decisive disadvantages: They are not transportable and are therefore unsuitable for field-testing or for athletes with diverse physical impairments. Furthermore, the existing devices do not replicate the athlete-chair unit, which is indispensable in wheelchair sports^6^. Sports scientists emphasize the need for testing in the athlete’s own chair to ensure the results can be transferred to real performance contexts^6,7^.

To address this gap, we developed a novel, portable trunk strength diagnostic device that provides a mobile, chair-integrated system for objective performance analysis and classification. This device enables the assessment of isometric trunk strength in four directions (flexion, extension, and bilateral lateral flexion) while the athlete remains seated in their wheelchair. This innovation has potential applications in performance diagnostics, training evaluation and equipment optimization (e.g. chair configuration)^6,7^.

Accordingly, the present study therefore aimed to: (I) examine the reliability of the newly developed device using a test-retest design; and (II) investigate its potential to discriminate between different functional classification levels in wheelchair basketball athletes.

We hypothesized that the device would demonstrate good-to-excellent test-retest reliability across all four directions, with acceptable measurement error quantified using established reliability metrics and reporting standards^24^. Further we hypothesized that athletes with higher functional classification levels would exhibit higher trunk strength compared with lower levels, consistent with the role of trunk impairment in classification and performance.

## Results

### Reliability measurement in able-bodied persons

**Fig. 1:**
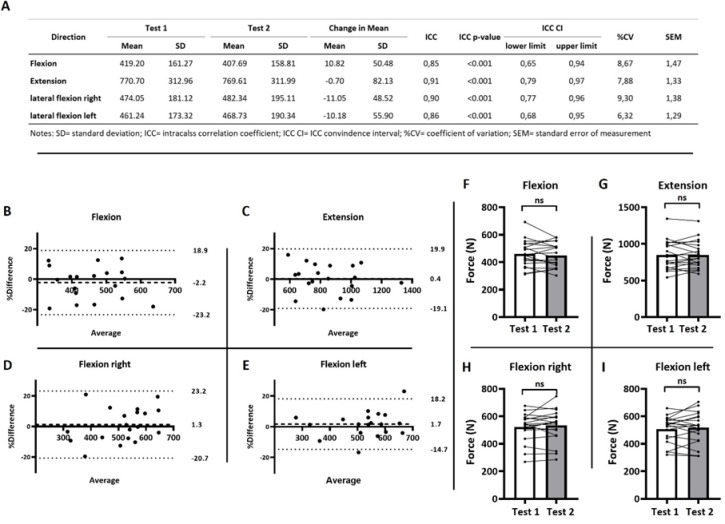
Reliability measures of able-bodied persons (AB-cohort; *n* = 19) in a wheelchair setting. (**A**) Tabular results of test-retest reliability measures for isometric strength assessment of the trunk. (**B**–**E**) Bland Altman plots (percentage difference: 100 x (test 2 – test 1)/average). Dashed line: mean bias; dotted lines: lower and upper limits of agreement. (**F**–**I**) Differences in maximal isometric trunk strength between test days 1 and 2. Paired t-tests, *p* < 0.05. ns = not significant.

For a first evaluation of the device, we tested able-bodied persons (AB) in a test-retest design to determine its reliability. A Shapiro–Wilk test of normality revealed a normal distribution for all variables in the AB-cohort. The intraclass correlation coefficient (ICC (3,1)) indicated significant reliability across all four movement directions (*p* < 0.001), with good to excellent agreement between both testing days (Fig. 1A). Specifically: flexion (ICC = 0.85, 95% CI: 0.66–0.94), extension (ICC = 0.91, 95% CI: 0.79–0.97), lateral flexion left (ICC = 0.86, 95% CI: 0.68–0.95), and lateral flexion right (ICC = 0.90, 95% CI: 0.77–0.96).

The coefficients of variation (CVs) were < 10% for all directions (Fig. 1A). Descriptive statistics for the forces generated in each direction (in Newton) are presented together with ICC, CV, and SEM values in Fig. 1A.

To further assess measurement consistency and to evaluate potential systematic bias, Bland–Altman analyses were performed. Percentage differences were calculated as 100 × (test 2 – test 1)/average, and plotted against the mean of the two tests (Fig. 1B-E).

When averaging across all four movement directions (Fig. 3), the mean bias was 0.3% (± SD: 1.7%). The standard deviation of the bias was 10.1% (± SD: 1.2%), and the average limits of agreement (LoA) ranged from + 20.1% to − 19.4% (± SD: 3.6% and 2.2%, respectively). Bland–Altman results for each direction are presented in Fig. 4B–E: Flexion: bias = − 2.17%, LoA = + 23.2% to − 18.9%; Extension: bias = 0.4%, LoA = + 19.9% to − 19.1%; Lateral flexion right: bias = 1.3%, LoA = + 23.2% to − 20.7%; Lateral flexion left: bias = 1.7%, LoA = + 18.2% to − 14.7%.

Consistent with the low bias observed, no statistically significant differences in maximal force production between the two test days were found across any of the four movement directions (Fig. 1F–I).

Reliability measurement in elite wheelchair basketball athletes.

**Fig. 2:**
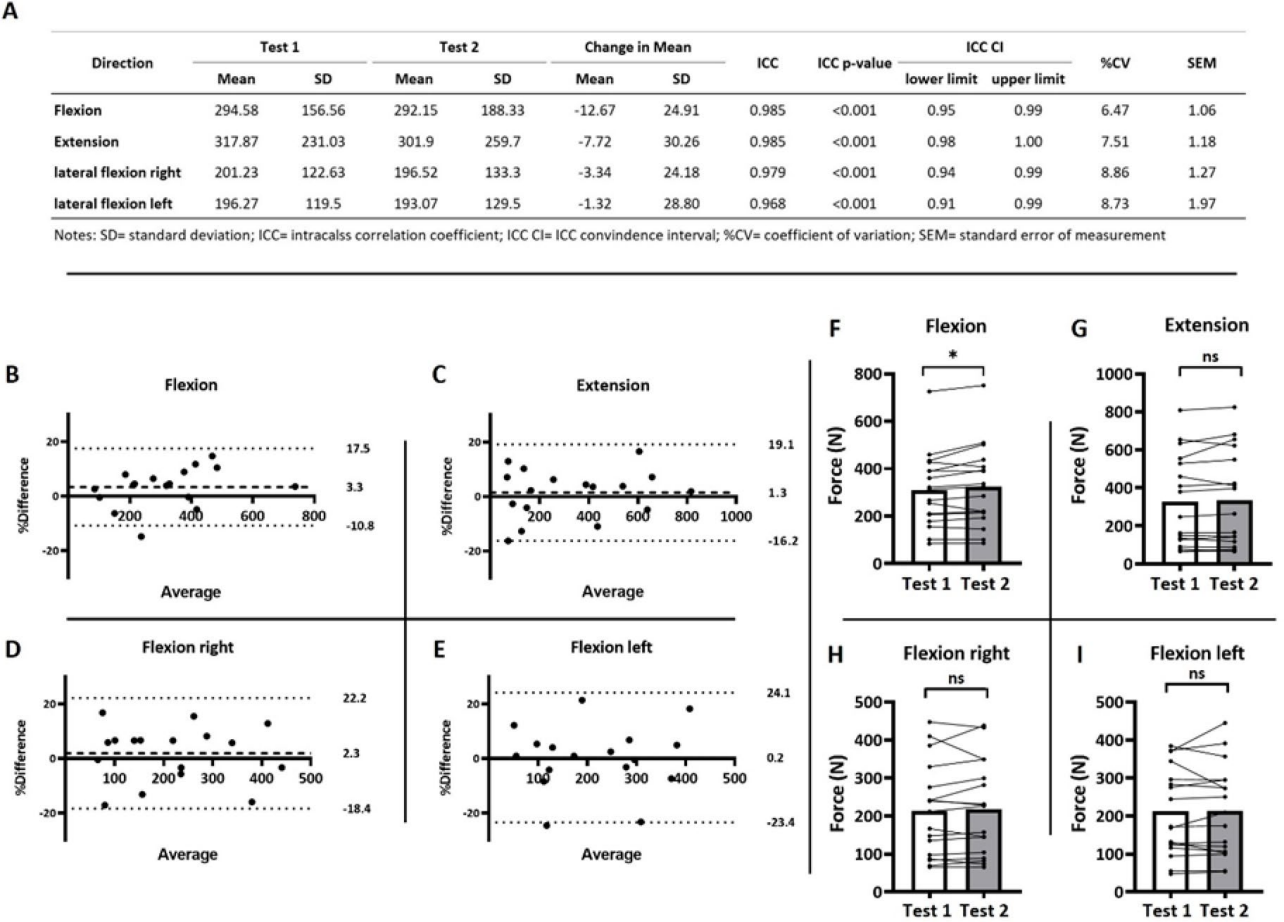
Reliability measures of elite wheelchair athletes (WC-cohort; *n* = 17) (**A**). Tabular results of test-retest reliability measures for isometric strength assessment of the trunk. (**B**–**E**) Bland Altman plots (percentage difference: 100 x (test 2 – test 1)/average). Dashed line: mean bias; dotted lines: lower and upper limits of agreement. (**F**–**I**) Differences in maximal isometric trunk strength between test days 1 and 2. Paired t-tests, *=*p* < 0.05. ns = not significant.

After testing the device with able-bodied participants, we repeated the measurements in elite WB athletes (Fig. 2).

A Shapiro–Wilk test of normality revealed a normal distribution for all movement directions except extension. The ICC (3,1) indicated significant reliability across all four directions (*p* < 0.001), with excellent agreement between the two testing days (Fig. 2A). Specifically: flexion (ICC = 0.99, 95% CI: 0.95–1.00), extension (ICC = 0.99, 95% CI: 0.98–1.00), lateral flexion left (ICC = 0.97, 95% CI: 0.92–0.99), and lateral flexion right (ICC = 0.98, 95% CI: 0.95–0.99).

The coefficients of variation (CVs) were < 10% for all directions (Fig. 2A). Descriptive statistics for the forces (in Newton) generated in each direction are presented together with ICC, CV, and SEM values in Fig. 2A.

As with the AB-cohort, Bland–Altman analyses were performed to assess consistency and potential systematic bias in the wheelchair-athlete cohort. Corresponding plots were generated by plotting the percentage difference (100 × (test 2 – test 1)/average) against the mean of the two tests (Fig. 2B-E).

When averaging across all four movement directions, the mean bias was 1.7% (± SD: 1.2%). The standard deviation of the bias was 9.7% (± SD: 2.1%), and the average limits of agreement (LoA) ranged from + 20.7% to − 17.2% (± SD: 3.9% and 5.2%, respectively). Bland–Altman results for each direction are presented in Fig. 4: Flexion: bias = 3.3%, LoA = + 17.5% to − 10.8%; Extension: bias = 1.4%, LoA = + 19.1% to − 16.2%; Lateral flexion right: bias = 1.9%, LoA = 22.2% to − 18.4%; Lateral flexion left: bias = 0.3%, LoA = + 24.1% to − 23.4%.

No statistically significant differences in maximal force production between the two test days were observed in three of the four movement directions (Fig. 2F-I). Only flexion showed a significant difference, with higher force production on the second testing day.

Testing for discriminatory ability of the device between different WB classification levels.

Next, we investigated whether the device can discriminate between different WB classification levels. For this purpose, a larger cohort than in the repeated-measurements analyses was tested (*n* = 55; 44 males, 11 females). However, the sample size still was insufficient to adequately represent all eight WB classifications for statistical analysis. Therefore, neighboring classifications were combined into four groups: Group I (classes 1.0 & 1.5): *n* = 13, Group II (classes 2.0 & 2.5): *n* = 12, Group III (classes 3.0 & 3.5): *n* = 23 and Group IV (classes 4.0 & 4.5): *n* = 7.

**Fig. 3:**
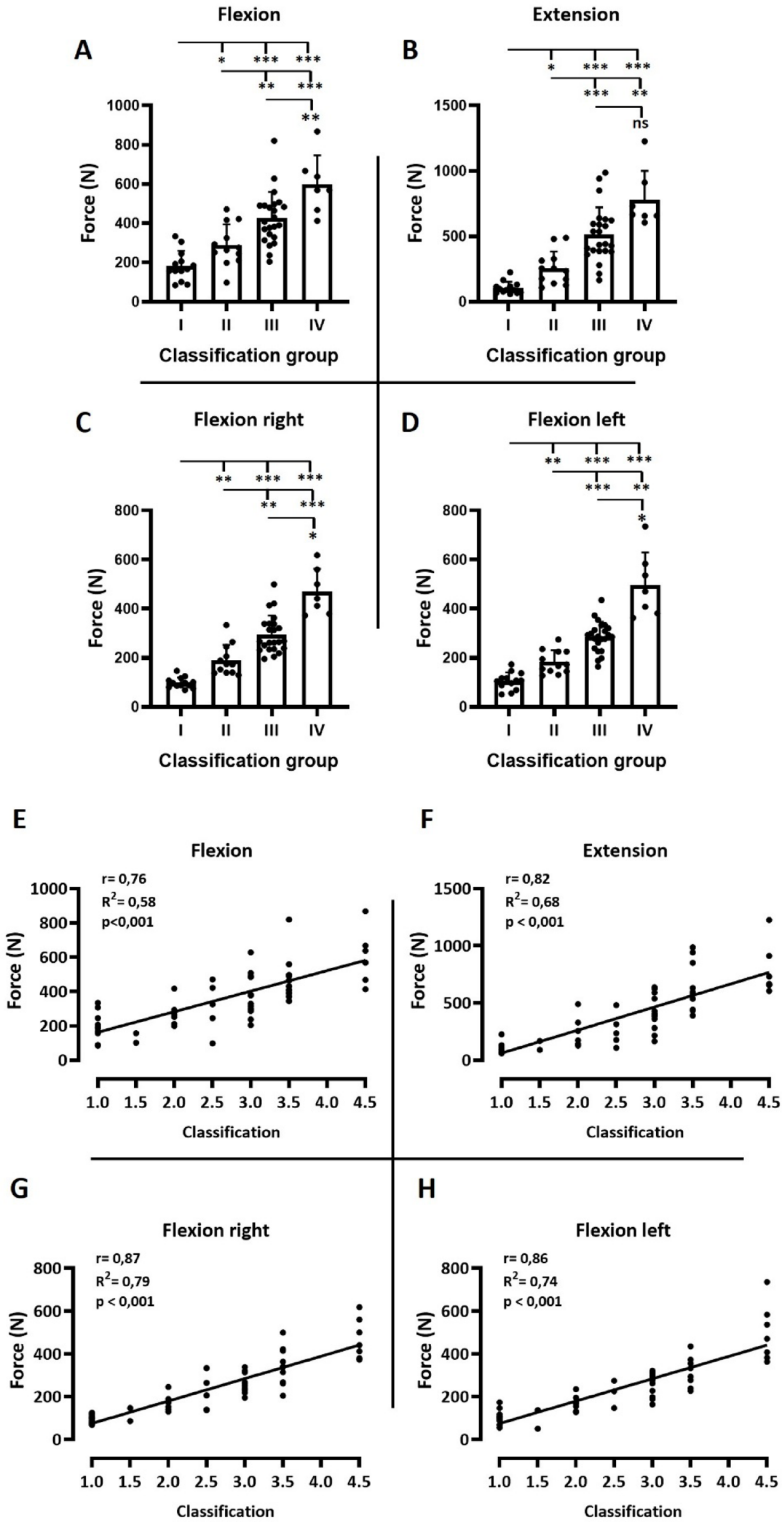
Discrimination between different classifications in wheelchair basketball athletes by exerted maximal isometric force. (**A**-**D**) Difference in exerted forces for the different directions between distinct classification-groups. Group I (classes 1.0 & 1.5): *n* = 13, Group II (classes 2.0 & 2.5): *n* = 12, Group III (classes 3.0 & 3.5): *n* = 23 and Group IV (classes 4.0 & 4.5): *n* = 7. Statistical methods: A) one-way ANOVA with Holm–Sidak’s post hoc test; *p* < 0.05. B-D) Welch ANOVA with Tamhane’s post hoc test; *p* < 0.05. *p < 0.05; **p < 0.01; ***p < 0.001. (**E**-**F**) Relationship between classification (1–4,5) level and force production in the different directions using Pearson’s correlation coefficients. 1: *n* = 11, 1.5: *n* = 2, 2: *n* = 7, 2.5: *n* = 5, 3: *n* = 13, 3.5: *n* = 10, 4.5: *n* = 7.

A Shapiro–Wilk test revealed normal distribution in all datasets except for extension and right lateral flexion. In addition, significant variance differences were observed in extension, right lateral flexion, and left lateral flexion (Brown–Forsythe test, *p* < 0.05). Accordingly, Welch ANOVA with Tamhane’s post hoc test was applied for these directions (*p* < 0.05), while flexion was analyzed using one-way ANOVA with Holm–Sidak’s post hoc test (*p* < 0.05; Fig. 3A–D).

Force production differed significantly between the classification-groups (I–IV) in all four directions (Fig. 3A–D). In each case, higher classification-groups achieved greater force values than lower groups. The only exception was extension, where group IV did not differ significantly from group III.

To examine the relationship between classification level and force production, Pearson’s correlation coefficients were calculated and visualized with linear regression (Fig. 3E–H). Here, each classification level was analyzed individually, without grouping. Very strong correlations were observed across all four directions (*r* = 0.76–0.87; R² = 0.58–0.79; *p* < 0.001).

Comparison of trunk force production between elite wheelchair basketball players and able-bodied persons.

**Fig. 4:**
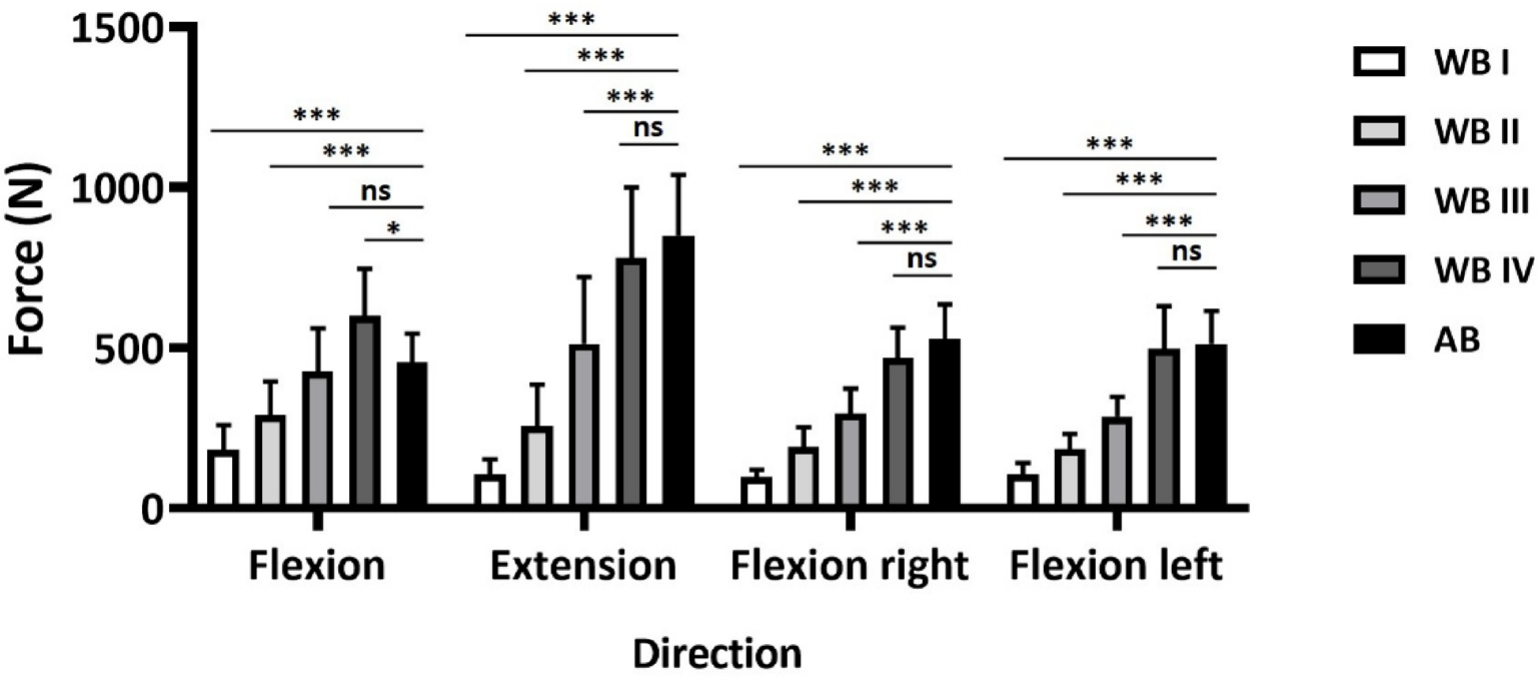
Comparison in exerted force between able-bodied persons (AB; *n* = 19) and wheelchair basketball athletes (WC; *n* = 55) of different classification-groups. Group I (classes 1.0 & 1.5): *n* = 13, Group II (classes 2.0 & 2.5): *n* = 12, Group III (classes 3.0 & 3.5): *n* = 23 and Group IV (classes 4.0 & 4.5): *n* = 7. Flexion was analyzed using one-way ANOVA with Holm–Sidak’s post hoc test, while the other directions were analyzed using Welch ANOVA with Tamhane’s post hoc test (*p* < 0.05). *p < 0.05; ***p < 0.001; ns = not significant.

Finally, we compared force production across the four movement directions between able-bodied persons (AB-cohort) and wheelchair basketball athletes of the different classification-groups (Fig. 4). For this analysis, the previously defined classification-groups (I–IV) were used, and the AB-cohort served as the control. Due to the aforementioned inconsistencies in normal distribution and differences in variances, flexion was analyzed using one-way ANOVA with Holm–Sidak’s post hoc test, while the other directions were analyzed using Welch ANOVA with Tamhane’s post hoc test (*p* < 0.05).

In all movement directions except flexion, the AB-cohort was significantly stronger than groups I, II, and III (*p* < 0.001), with no significant difference compared to group IV. For flexion, the AB-cohort was stronger than groups I and II (*p* < 0.001), showed no significant difference to group III, and was outperformed by group IV (*p* < 0.05).

## Discussion

The purpose of the present study was to evaluate the reliability of a newly developed device for trunk strength diagnostics in wheelchair athletes and to assess its potential to discriminate between functional classifications.

The main findings of this study were as follows: (1) The device demonstrated good to excellent ICC reliability in both, able-bodied (Fig. 1A) and wheelchair athletes (Fig. 2A), (2) Coefficient of variation indicates tolerable variance of < 10% (Fig. 1A and 2A), (3) Bland-Altman analyses indicated a low bias and tolerable limits of agreement (Figs. 1B-E and 2B-E), and (4) The device successfully discriminated between different classification-groups (I-IV) in wheelchair basketball, additionally showing high correlations between individual classifications (1–4,5) and exerted force (Figs. 3 and 4).

Although the present study focused on wheelchair basketball athletes, the device can be applied to a wide range of wheelchair-based sports like wheelchair-rugby, wheelchair-tennis or wheelchair-badminton. With minor modifications, the device could even be adapted for disciplines such as sledge hockey or para-rowing. A salient feature of the device is its transportability, which allows field-based testing directly in the athlete’s own wheelchair, thereby treating the athlete – chair combination as a functional unit.

Trunk strength testing in able-bodied populations has been extensively studied, with a wide variety of methods, including isokinetic^9,25^ and isometric dynamometers^9,26,27^, hand-held dynamometers^25,28–30^, and field-based core stability tests^31^. Reported test-retest reliabilities in these settings are generally high, with intraclass correlation coefficients (ICC) ranging from good (0.85) to excellent (0.96)^9,26,28,30^. In contrast, trunk strength assessment in wheelchair athletes remains challenging. Only a few studies addressed this population, with the majority focusing on indirect measures such as functional reach^32^, seated balance^5^, or propulsion mechanics^1,4,20–22^. Where direct strength testing was attempted, stationary isokinetic devices were typically used, which are not transportable and particularly, cannot account for the athlete-chair interaction^5^.

Our approach differs substantially in that the device is transportable and allows testing directly in the athlete’s own sports chair, treating the athlete and its wheelchair as one functional unit. The ICC values obtained in our study were consistently in the good-to-excellent range, in line with trunk strength tests using laboratory-based devices like the IsoMed in able-bodied cohorts^9^ and with handheld protocols under optimized stabilization^28^.

The coefficients of variation observed in our study ranged from 6.32% to 9.3%, which is consistent with the range reported in controlled laboratory assessments of trunk strength, which typically range from 5–12%^8,9,28^. While strength and jump field-tests for able-bodied athletes commonly used for performance diagnostics mostly demonstrate a CV under 10%^31^, studies using handheld dynamometers in less standardized field settings reported substantially higher variability with a relative typical error of over 13%^25^. Consequently, our findings indicate that the variability of our device is comparable to laboratory-based protocols, despite its application in field and sport-specific contexts.

Bland-Altman analyses further corroborated these findings, demonstrating no relevant systematic measurement error. The mean bias was close to zero across all directions (0.3% for the WC-cohort and 1.7% for the AB-cohort), indicating excellent overall agreement between test and retest. The limit of agreement (LoA) ranged from − 2.2 to + 1.7% for the wheelchair athletes and from − 0.3 to + 3.3% for the able-bodied participants. Contrary to the majority of previous studies that reported absolute values for reliability indices, we expressed our data as relative values in percent. This approach is notable for its consideration of high inter-individual strength discrepancies, which are apparent in all para-sport cohorts like WB, where the degree of impairment exhibits significant force variation across classification levels. While the mean bias between test and retest was negligible, the LoA were relatively wide (approximately − 20 to + 20%). This finding suggests that the device offers adequate agreement and consistency at the group level. Nevertheless, it is critical to acknowledge that a certain degree of variability occurs at the individual level. The relative LoA observed in our study are considered acceptable for field-based diagnostics and are consistent with the ranges reported in previous studies. For instance, Croci et al. (2023) reported seemingly negligible absolute LoA of -3.5 to + 3.8 Nm for Biodex dynamometer testing of shoulder strength in an able-bodied cohort. However, when expressed as relative values, these correspond to approximately − 9 to + 10.5% for internal rotation and as much as -20 to + 30% in abduction. A more field-based approach, using functional electromechanical dynamometry, yielded relative LoA in the range of -20 to + 15%^27^. These comparisons underline that the variability observed with our device is consistent with both laboratory- and field-based reliability data.

Despite the overall general positive reliability indices, a considerable degree of individual variability remained. Two primary factors are likely to account for this: First, a familiarization effect was observed via Bland-Altman, as most participants achieved higher values in the second session and reported that the initial trial felt unusual due to the fixation. Second, the fixation itself, especially the chest harness, which needed to be tightened firmly to prevent soft tissue displacement and changes in the line of pull. In this study, a standard climbing harness was used. However, the development of a trunk-specific harness or vest could potentially reduce measurement error and further improve reliability.

Overall, the results indicate that our device attains a reliability that lies between other field-used and that of gold-standard laboratory systems, while offering the added benefits of transportability, field applicability, and sport-specific, in-chair testing.

Previous literature has emphasized the importance of trunk strength for athletic performance of able-bodied athletes, as trunk strength training has been shown to enhance athletic performance in a variety of sports^16^ like basketball^17^, football^18^ or hockey^19^. In wheelchair sports, trunk motion and control strongly influence propulsion^20,22,33^ and maneuvering and serves as the foundation for classification process^2,4,5^.

We tested for differences in trunk strength across wheelchair basketball classification levels and observed, unsurprisingly, a systematic increase in force production with increasing classification level, which is consistent with previous findings that trunk strength and control correlate positively with classification^1,2,4,5,10^. These results are also consistent with evidence from wheelchair rugby, where systematic differences in acceleration and sprint performance could be explained by the degree of trunk impairment^4^. It can be argued that, in principle, the device does not measure the pure trunk strength because due to the setup, force is generated by the interaction of synergistic muscles along the kinetic chain, not exclusively by the trunk muscles. The observed differences are not only due to lower force capacity of lower classification levels or reduced force capacity caused by impairment, but also due to the fact that WB athletes in higher classes are able to utilize their legs and hips. For instance, during trunk flexion, athletes in higher classification levels are able to engage the hip flexor muscles to assist force generation. However, this is irrelevant in terms of performance diagnostics for wheelchair sports, as the athlete and the wheelchair must be regarded as a functional unit. For this reason, participants were deliberately instructed to generate maximal force, allowing the use of the hip flexors or extensors if their functional abilities permitted. This approach ensured that the measurement reflects the athlete’s overall capacity to exert force from the seated position. Accordingly, the device has been engineered to assess the muscle groups that enable the athlete to perform flexion, extension and lateral movements within the chair, providing mobility and stability during play.

Thus, our data confirm the central role of trunk function in the classification of wheelchair basketball players and reinforce calls for evidence-based approaches for classification which were formulated by Vanlandewijck et al. (2010, 2011) and Altmann et al. (2018). Furthermore, the strong correlations between classification and maximal isometric trunk strength found in our data (Fig. 3E-H) suggest that our device could provide an objective measurement of the forces an athlete can exert in different directions on the playing field in their wheelchair. This could serve as an additional tool to existing classification procedures, which currently rely heavily on observational assessments.

When comparing trunk strength across classification-groups with the able-bodied cohort, the able-bodied athletes consistently produce higher forces, with the exception of classification-group IV, where no significant differences were found (Fig. 4). This finding aligns with the anticipated correlation between the severity of impairment and the individual’s force-generating capacity^1,2,5^. Interestingly, however, in trunk flexion, no significant differences were found between WB athletes of class III and the able-bodied cohort, whereas class IV WB athletes exhibited maximal forces that even surpassed those of their able-bodied counterparts (Fig. 4). This atypical pattern may be partly explained by the fact that athletes in classes 4.0 and 4.5 are only minimally impaired, with limitations usually affecting the lower limbs, rather than the trunk. An additional rationale for this phenomenon may be related to sport-specific adaptations in WB: trunk flexion is used frequently in wheelchair propulsion, ball handling and maintaining forward stability during high-intensity play, as confirmed by propulsion mechanics and kinematic studies^6,20,22,33^. In contrast, physically trained, able-bodied participants may not rely on trunk flexion to the same extent in daily locomotion or sports. These findings highlight the unique trunk strength profile of wheelchair basketball athletes and emphasize the importance of sport-specific diagnostics.

Conducting trunk strength assessments in the athlete’s own wheelchair ensures sport-specific validity and direct transfer to performance contexts. The device provides a means of monitoring the effects of trunk-specific training, optimizing chair configurations^6^ and contributing to objective classification research. Its portability also enables it to be used in field testing, complementing current practices for performance testing in wheelchair court sports^7^. Of particular note, the device is currently implemented as a component of the routine performance screening of the German national wheelchair basketball team, indicating its immediate applicability and practicability within high-performance settings.

The following limitations of the study have to be mentioned. The unequal distribution of sex across the wheelchair cohorts, as well as the lack of matching sex ratios between the wheelchair and able-bodied groups, may limit the generalizability of class-specific reference values. Furthermore, the analysis of both men and women within each group was done collectively rather than separately by their sex. While the primary objective centered on enhancing device reliability and demonstrating classification-level discrimination, future research endeavors should include the recruitment of sex-balanced cohorts and/or the explicit modeling of sex effects (e.g., stratified analyses or incorporating sex as a covariate) during the derivation of normative values.

Furthermore, the exclusion of attempts employing a 1.5 standard deviation rule (see Methods) was implemented as a pragmatic quality-control measure to eliminate implausible trials. As this criterion does not represent a consensus standard, it is not possible to fully rule out a minor influence on estimated strength values.

Follow -up studies might use the device to assess the effects of training interventions on trunk strength in wheelchair-based sports. Furthermore, the device may support the development of evidence-based classification criteria by providing quantitative measures of trunk function. In a broader context, the device might be used in older adults or non-sport populations with impairments, where multidirectional isometric trunk strength in a seated position is of interest, or for example to assess trunk strength during the initial phase of a sit-to-stand task.

In summary, this study demonstrates that the examined trunk strength diagnostic device for wheelchair users is both reliable and sport-specific. It enables the objective assessment of in-wheelchair trunk strength, distinguishes between classification-groups and may in future provide valuable insights for training monitoring, equipment optimization and classification research. The device enables a significant step towards more evidence-based and sports-specific diagnostics in wheelchair sports.

## Methods

### Participants

For the study, three cohorts were included. Two of them performed test-retest measurements and a third cohort was assembled to evaluate the discriminatory capacity of the device. Ensuing, these cohorts are specified in more detail.

For the test-retest measurements, able-bodied (AB) persons as well as disabled wheelchair basketball (WB) athletes were recruited. They were separated into two different cohorts: (1) AB-cohort, consisting of sports science students which were physically active, but not specialized to strength dominated disciplines (*n* = 19, 9 males and 10 females; age: 24.5 ± 4.2 years; body mass 71.8 ± 9.7 kg; height 172.6 ± 8.0 cm; mean physical activity volume 10.4 ± 5.6 h/week). (2) WB cohort, consisting of persons participating in elite WB at international level (*n* = 17, 12 males, 5 females; 28.4 ± 5.9 years; 73.6 ± 19.7 kg). To evaluate the discriminatory capacity of the device, a third cohort was included. This consisted of the WB cohort (*n* = 17) which was extended with 38 further athletes who were tested at only one occasion (*n* = 55, 44 males, 11 females; 27.4 ± 6.9 years; 68.6 ± 16.7 kg).

Participant characteristics for all three cohorts are displayed in Table 1A and B presents the number of participating wheelchair basketball athletes in each International Wheelchair Basketball Federation (IWBF) classification level^11^.

**Table 1:**
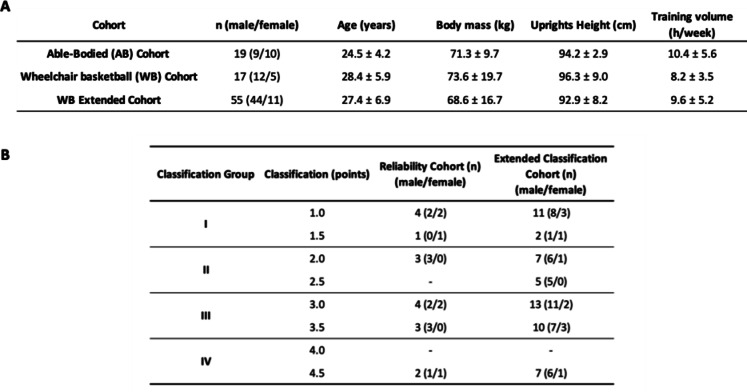
Descriptive characteristics of the examined cohorts and Participant distribution by International Wheelchair Basketball Federation classification level for the wheelchair reliability- and extended classification-cohort.

### Measurement device

**Fig. 5:**
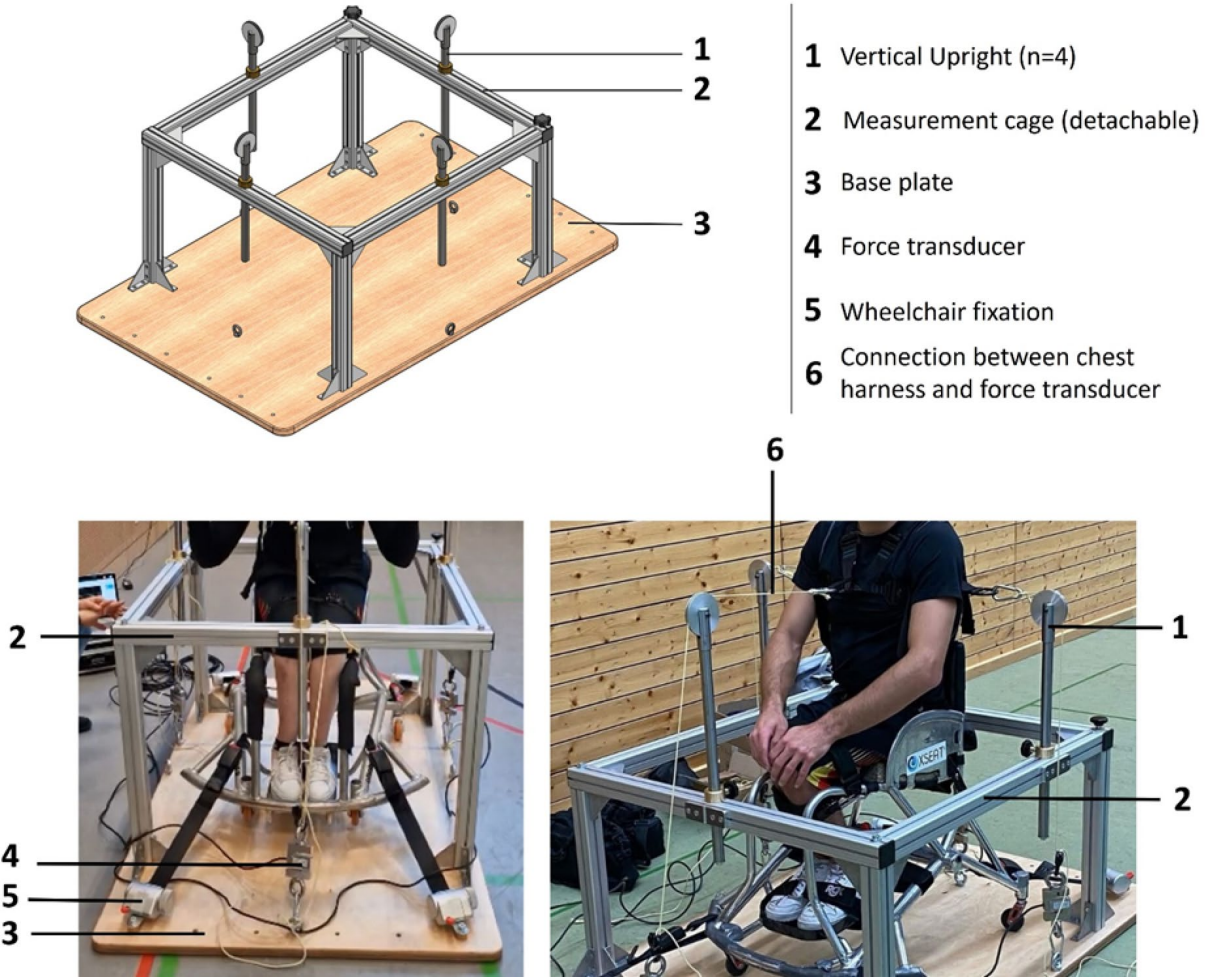
Schematic representation of the measurement device and photographs illustrating the experimental setup during testing.

The diagnostic device was manufactured in the precision engineering workshop of the German Sport University Cologne. It consists of a solid wooden base plate measuring 1.0 × 1.2 m and 2 cm in thickness, which has a modular, detachable aluminum frame (measurement cage) consisting of four height-adjustable vertical steel uprights connected by horizontal crossbars (Fig. 5).

The front crossbar can be removed to allow barrier-free access for wheelchair users. Before measurement, wheelchairs are secured to the base plate using tension straps, and participants wear a chest harness that is connected via inelastic ropes to force transducers (Mod. CS150-3Q1, SAUTER GmbH) which are connected to a laptop, where force curves are displayed and data extracted using BlueDAQ software (Version 2–02; https://www.interfaceforce.de/messverstaerker/bluedaq/). Pulley-based rope guidance is provided to ensure that the wheelchair remains completely immobile during testing, guaranteeing accurate measurements.

Force sensors are positioned on each side of the base plate to capture isometric strength in four directions: trunk flexion (anterior placement), trunk extension (posterior placement) and lateral flexion to the left and right (lateral placements). Signals were recorded in real time, with force expressed in newtons (Fig. 5).

### Testing protocol

All tests were conducted between 09:00 AM and 02:00 PM either at the Olympic Training Centre NRW/Rhineland or in the training facilities of the respective wheelchair basketball teams. Participants were instructed to avoid intense physical activity and strength training for a minimum of 24 h prior to testing. To standardize nutritional status, participants were instructed to perform the test in a postprandial state, this is to say, after having consumed their last meal at least two hours before testing. It was requested that all participants continue to maintain their usual hydration routines.

Prior to testing, participants performed a standardized warm-up consisting of three submaximal isometric contractions at approximately 50%, 70% and 90% of their perceived maximum effort for each direction of movement. Each contraction was gradually built up over 2–3 s and held until a clear force plateau was reached. Standardized rest periods of 30 s were provided between individual maximal contractions.

The subsequent maximal strength testing followed a fixed sequence: flexion, extension, lateral flexion to the left and lateral flexion to the right. Three maximal trials were performed for each direction, separated by 30 s of rest. Participants were instructed to place their hands on their temples with their elbows rotated outwards to standardize posture. Each maximal effort was sustained until a plateau was observed in the force curve.

To replicate the sport-specific situation, wheelchair basketball athletes were measured while being strapped in their sports chairs as is customary. Able-bodied participants were seated and strapped up in a test wheelchair with additional padding around the pelvis and lumbar spine to ensure stability and reduce extraneous movement.

### Positioning, fixation and instructions

The wheelchair was positioned centrally on the base plate and aligned such that the rear wheels were parallel to the vertical uprights of the measurement cage. A visual inspection was conducted to verify the alignment and ensure a symmetrical setup to avoid lateral forces on the frame. The vertical uprights were adjusted individually to the height of the chest harness, thereby ensuring consistent rope angles across athletes, i.e.: ropes run horizontally from the pulleys to the attachment points of the harness, respectively.

The line of pull was aligned with the anatomical axis of movement (anterior/posterior for flexion/extension, lateral for lateral flexion). This ensured that the resulting force vectors corresponded to the intended trunk movement directions.

The chest harness was positioned across the sternum and lower rib cage, thus ensuring that respiration was not restricted while ensuring firm fixation. Care was taken to minimize slack in the straps and simultaneously avoid excessive compression of the thorax to ensure participant comfort and reproducibility.

Participants received standardized instructions to generate maximal force in the specified direction for approximately three seconds, avoiding any jerking or compensatory movements. They were explicitly instructed to exert as much force as possible from their seated position, allowing activation of the hip flexors or extensors when their level of impairment permitted. During the test, participants were instructed to position their hands at the temples with the elbows rotated outward to prevent the use of the upper limbs and to maintain a consistent posture across trials.

During all maximal trials, participants received consistent verbal encouragement from the investigator to ensure maximal effort, with the phrasing and intensity of the encouragement being uniform across all participants and trials.

### Data processing and statistical analysis

For each trial, force-displacement curves were recorded using BlueDAQ software (Version 2–02; https://www.interfaceforce.de/messverstaerker/bluedaq/). Peak force values (N) were extracted using a custom algorithm written in R programming language. (Version 2024.12.0 + 467; Posit PBC) algorithm. The force-time curves, recorded at a sampling frequency of 500 Hz, were filtered using a moving average to smoothen the signal. Subsequent to the identification of the peaks by the algorithm, a visual inspection was conducted to ensure plausibility and to exclude artifacts. This process enabled the exclusion of instances where participants appeared to utilize momentum rather than generating a legitimate isometric contraction. In the context of the study, outliers were systematically eliminated from consideration for each direction when deviating more than 1.5 standard deviations from the mean of the trials. Subsequently, a new mean of the remaining trials per direction was then calculated for each session.

All data were tested for normal distribution using the Shapiro-Wilk test. When data was normally distributed, paired t-tests were used to identify systematic differences between test and retest measurements, while one-way ANOVA was applied for group comparisons between WB classifications.

Reliability was assessed by calculating intraclass correlation coefficients (ICC) and coefficients of variation (CV). Further, agreement between sessions was analyzed using Bland-Altman plots to determine mean bias and limit of agreements. ICC values were interpreted according to Koo and Li (2016)^24^. For comparisons between WB classifications and able-bodied groups, one-way ANOVA was applied. The homogeneity of variances was verified using Brown-Forsythe test. When the assumption was met (trunk flexion), Holm-Sidak’s multiple comparison test was performed. When normality or homogeneity were violated (all other directions), Welch’s ANOVA with Tamhane’s multiple comparisons test was applied. The significance level was set at *p* < 0.05.

## Data Availability

The data presented in this study are available on request from the corresponding author.
